# The Effect of *Moringa oleifera* Leaf Extract on C2C12 Myoblast Proliferation and Redox Status Under Oxidative Insult

**DOI:** 10.3390/antiox13121460

**Published:** 2024-11-28

**Authors:** Roberta Ceci, Mariateresa Maldini, Piergiorgio La Rosa, Laura Sireno, Cristina Antinozzi, Mark E. Olson, Ivan Dimauro, Guglielmo Duranti

**Affiliations:** 1 Laboratory of Biochemistry and Molecular Biology, Department of Movement, Human and Health Sciences, Università degli Studi di Roma “Foro Italico”, Piazza Lauro De Bosis 6, 00135 Roma, Italy; 2 SCIEX Italia S.r.l., Via Montenapoleone, 8, 20121 Milano, Italy; mariateresa.maldini@sciex.com; 3 Division of Neuroscience, Department of Psychology, Sapienza University, Via dei Marsi 78, 00185 Rome, Italy; piergiorgio.larosa@uniroma1.it; 4 Laboratory of Biology and Human Genetics, Department of Movement, Human and Health Sciences, Università degli Studi di Roma “Foro Italico”, Piazza Lauro De Bosis 6, 00135 Roma, Italy; l.sireno@studenti.uniroma4.it (L.S.); ivan.dimauro@uniroma4.it (I.D.); 5 Laboratory of Endocrinology, Department of Movement, Human and Health Sciences, Università degli Studi di Roma “Foro Italico”, Piazza Lauro De Bosis 6, 00135 Roma, Italy; cristina.antinozzi@uniroma4.it; 6 Instituto de Biología, Universidad Nacional Autónoma de México, Tercer Circuito de CU S/N, Mexico City 04510, Mexico; molson@ib.unam.mx

**Keywords:** *Moringa oleifera* leaf extract (MOLE), C2C12 skeletal muscle cells, myoblast proliferation, glutathione, redox homeostasis, oxidative stress, LC-MSMS ZenoTOF 7600 system metabolomics analysis

## Abstract

Skeletal muscle tissue can regenerate after damage through the action of satellite cells, which proliferate as myoblasts when activated. Oxidative stress, marked by high rates of reactive oxygen species (e.g., hydrogen peroxide, H_2_O_2_), impairs this process by increasing myoblast cell death. *Moringa oleifera* leaf extract (MOLE), known for its antioxidant properties, was tested for its protective effects on C2C12 myoblasts under oxidative stress. We assessed MOLE’s impact on total antioxidant capacity (TAC), glutathione homeostasis (GSH/GSSG), cell viability, and wound recovery. The metabolomic analysis of MOLE using an LC-MSMS ZenoTOF 7600 mass spectrometry system identified key compounds, including peculiar glucosinolates (42.1%) and flavonoids (18.8%), as well as phenolic acids (4.5%) and other significant metabolites (34.6%; among them, amino acids, vitamins, and fatty acids). H_2_O_2_ disrupted myoblast redox balance and caused cell death, but MOLE treatment restored the GSH/GSSG ratio, improved TAC, and increased cell viability. Additionally, MOLE promoted faster wound closure in myoblasts exposed to H_2_O_2_. These findings suggest that MOLE can protect C2C12 myoblasts by restoring redox balance and enhancing recovery under oxidative stress.

## 1. Introduction

Skeletal muscle constitutes up to 50% of total body mass and plays an important role in maintaining optimal energy expenditure across the entire organism [1]. Rapid changes in fuel selection are a hallmark of metabolically healthy muscle. Consequently, preserving healthy skeletal muscle mass is essential throughout life in preventing or mitigating possible metabolic diseases. 

Skeletal muscle can regenerate through the activation of stem cells (satellite cells) by environmental signals [2]. Once activated, satellite cells exit cell-cycle arrest, proliferate as myoblasts, and form multinucleated cells with a complex process of cell alignment and fusion that is able to repair damaged sections of the myofibers and maintain muscle mass [3,4]. This is a complex process involving many cellular changes, and defects in the activation or proliferation of myoblasts can profoundly impair muscle regeneration [5,6,7]. In fact, a reduction in the regenerative potential of the fibers characterizes several physio-pathological conditions. Muscle mass loss (atrophy) and ALS (amyotrophic lateral sclerosis) are some examples of pathological conditions that involve the failure to activate myoblasts and severely affect human ability and health [8,9,10,11]. 

Among the molecules that profoundly influence muscle regeneration, reactive oxygen species (ROS), such hydrogen peroxide (H_2_O_2_), play a critical regulatory role [12,13,14]. Low-to-moderate ROS levels are essential for muscle adaptation, cell signaling, the regulation of gene expression, and muscle growth [13,15]. However, when ROS are produced at high levels, they lead to the oxidation of macromolecules, which results in impaired myoblast function, increased cell death, and hindered muscle repair. Elevated ROS levels occur under physiological conditions such as aging, or are associated with metabolic diseases like diabetes, as well as conditions of muscle mass loss, such as atrophy and sarcopenia [16]. 

The metabolic activity of muscles, which occurs in many types of physical exercise, especially intense or unaccustomed, leads to an increase in reactive oxygen species, and consequently, in oxidative stress, as indicated by elevated biomarkers of molecule oxidation in both skeletal muscle and blood tissues [15,17,18]. Additionally, prolonged periods of muscle disuse may induce processes that promote ROS production in skeletal muscle fibers [19]. 

On the other hand, an increase in oxidative stress can compromise muscle functionality and the ability to regenerate after damage, which can result in vigorous contraction. The ability of satellite cells to repair muscle damage and facilitate recovery can be severely impaired by ROS-induced molecular and cellular damage [16,20,21]. While low-to-moderate levels of ROS are essential for muscle adaptation, high levels of ROS significantly impair muscle function [22].

In this context, there is an ever-increasing demand for strategies that enhance physical performance by preventing muscle damage or aiding recovery after exertion. Among these, nutritional strategies to mitigate or reduce the damage caused by elevated ROS levels to macromolecules are the subject of current research [23,24].

In recent years, the natural products derived from *Moringa oleifera* Lam. (family Moringaceae; order Brassicaceae) have been the subject of numerous studies comprising different experimental models aiming to evaluate their antioxidant properties [25,26,27,28,29]. Different parts of the plant (i.e., leaves, seeds, roots) contain numerous bioactive molecules with antioxidant, antimicrobial, and/or anti-carcinogenic properties [30,31]. Extracts from *Moringa oleifera* demonstrate activity due to the presence of bioactive molecules such as isothiocyanates, tannins, saponins, flavonoids, and terpenoids, especially in the leaves [30,31,32,33]. Due to the remarkable efficacy of these bioactive molecules, and especially some unique isothiocyanates, *Moringa oleifera* is gaining global interest for a wide range of applications. Among the notable glucosinolates/isothiocyanate present, moringin, a potent isothiocyanate produced by the hydrolysis of the glucosinolate glucomoringin by the enzyme myrosinase, exhibits potent anti-inflammatory and indirect cytoprotective antioxidant activity, supporting the therapeutic use of *Moringa oleifera* extracts for various pathologies [33,34,35].

Moreover, flavonoids, other glycosides, and phenolic acids (e.g., chlorogenic acid, ferulic acid) are present in moderate concentrations in *Moringa oleifera* leaf extracts. These molecules have proven to be effective in counteracting the increase in reactive oxygen species [36,37,38,39], acting as primary antioxidants by either inactivating lipid free radicals or preventing the decomposition of hydroperoxides to produce free radicals [40,41,42]. 

Recently, our group demonstrated that *Moringa oleifera* leaf extract (MOLE) improved the capacity of C2C12 myotubes to respond to oxidative stress via the activation of the SIRT1-PPAR pathway [43]. Moreover, MOLE was able to induce the nuclear factor erythroid 2-related factor (Nrf2) and its target gene heme oxygenase-1 (HO-1) pathway [29], resulting in an increase in the antioxidant system of skeletal muscle cells. This restored the redox balance (assessed by total free thiols, Trx, and GSH/GSSG ratio increase levels) and increased the antioxidant enzymatic system (catalase (CAT), superoxide dismutase (SOD), glutathione proxidase (GPx), and glutathione S-transferase (GST)), significantly reducing markers of cellular oxidative damage (thiobarbituric acid reactive substance (TBAR) and carbonylated protein (PrCAR)) [44]. Based on these findings, we hypothesize that MOLE could efficiently protect C2C12 myoblasts subjected to a pro-oxidant environment. 

MOLE extract was analyzed for metabolic qualitative profiling by ultra-high-performance liquid chromatography–quadrupole time-of-flight mass spectrometry (UHPLC/QTOF-MS), as previously described [45]. 

The aim of this research was to provide scientific evidence about the effect of *Moringa oleifera* leaf extract on restoring redox balance in myoblasts subjected to an oxidative insult mimicking (for example) the high stress condition that occurs during intense muscle contractions. To this end, proliferative C2C12 myoblasts were treated with MOLE and/or exposed to 0.3 mM H_2_O_2_ in single and combined treatments for 24 h and then analyzed for (a) cell viability; (b) GSH homeostasis; (c) total antioxidant capacity (TAC); and (d) the ability of myoblasts to replicate and migrate in the culture plate under different experimental conditions through the “wound closure assay”. 

## 2. Materials and Methods

The chemical reagents used in this research were purchased from Sigma-Aldrich (St. Louis, MO, USA), unless otherwise specified.

### 2.1. MOLE: Ethanolic Extract of Moringa oleifera Leaves

The ethanolic extract of *Moringa oleifera* leaves was prepared as previously described [44]. Briefly, *Moringa oleifera* leaf powder (PureBodhi Nutraceuticals Ltd., London, UK) (1 g powder/10 mL ethanol 100%) was gently sonicated (Vibra-Cell CV 18 SONICS VX 11, Sonics & Materials, Newtown, CT, USA) twice for 10 min at +4 °C. After centrifugation (2000× *g* for 10 min at +4 °C), the resulting supernatant was collected and stored at −20 °C (stock solution equivalent to 15 mg/mL of dried leaves).

### 2.2. MOLE Qualitative Profiling

MOLE metabolomic qualitative profiling was carried out via ultra-high-performance liquid chromatography–quadrupole time-of-flight mass spectrometry (UHPLC/QTOF- MS) on a SCIEX ZenoTOF 7600 mass spectrometer (AB SCIEX GmbH, Landwehrstraße 54, Darmstadt, Germany), as previously described [43,45]. For deeper qualitative profiling, a sample was acquired using the approaches of Data-Dependent Acquisition (zeno DDA) and Data-Independent Acquisition (zenoSWATH). The obtained data were processed using SciexOS Software 3.3 (AB SCIEX GmbH, Landwehrstraße 54, Darmstadt, Germany) and the SCIEX Natural Products 2.1 Library (AB SCIEX GmbH, Landwehrstraße 54, Darmstadt, Germany) and NIST 2017 Library in order to search for compound spectra databases.

The Formula Finder and Chemspider tools in SciexOS software (SciexOS software version 3.3) were used for the analysis of molecules that had no correspondence with the library but had accurate TOF MS and TOF MS/MS spectra. ZenoSWATH allowed for more confident identification, particularly for cases of a very low abundance of secondary metabolites. This method enhances the detection of these very-low-abundance metabolites by searching for precursor ions and detecting their diagnostic fragment ions through accurate mass measurements [43,45].

### 2.3. Cell Cultures 

Mouse C2C12 myoblasts (ATCC, Manassas, VA, USA) were cultured in sterile conditions at 37 °C with 5% CO_2_ in a humidified atmosphere at a density of 2 × 10^3^/cm^2^ in Dulbecco’s modified Eagle’s medium (DMEM; HyClone, Oud-Beijerland, The Netherlands). The media were supplemented with Glutamax-I (4 mM l-alanyl-l-glutamine), 4.5 g/L glucose (Invitrogen, Carlsbad, CA, USA), and 10% heat-inactivated fetal bovine serum (Hy-Clone, Oud-Beijerland, The Netherlands). Cells were split 1:6 twice weekly and fed 24 h before each experiment, as described previously [44,46]. 

### 2.4. Cell Viability

C2C12 viability was assessed by the Trypan blue exclusion assay, as previously described [47]. Briefly, cells were seeded at low density (<15%) in 25 cm^2^ culture flasks. After 24 h (≈25% confluence), they were treated with different concentration of H_2_O_2_ (0.1–1 mM) (H_2_O_2_ dose dependence), and various dilutions of the MOLE stock solution (1:50–1:1000 working solution) for 24 h (MOLE dose dependence). Additionally, different H_2_O_2_ exposure times (6–48 h, time-dependent) were evaluated. Cells reached a maximum confluence of 75%. Accordingly, for the combined treatments, concentrations of 0.3 mM H_2_O_2_ and a 1:100 dilution of MOLE were chosen to assess their effects on myoblast viability and oxidative stress responses. Under these experimental conditions, the ethanol concentration in the working solutions (0.1%, *v*/*v*) did not affect myoblast viability. After the treatments, cells were trypsinized and collected by centrifugation (1200× *g* for 10 min at room temperature), and then cell viability was assessed using the Trypan blue exclusion assay (cells/dye 0.05% *v*/*v* solution in PBS mixed in a ratio of 1:1). With this method, it is possible to count the live cells that have rejected the Trypan blue dye (alive) from the colored cells that have incorporated it (dead cells) by analyzing the sample on a hemocytometer. Experiments were performed in triplicate with different cell preparations and the results are expressed as the number of cells counted. 

### 2.5. Trolox^®^ Equivalent Antioxidant Capacity

Cellular total antioxidant capacity was evaluated as previously described [29,48]. The Trolox^®^ equivalent antioxidant capacity (TAC) assay evaluates the ability of samples (cell lysates) to prevent ABTS+ radical formation compared to Trolox^®^ (vitamin E analogue) standards in a pro-oxidative buffer. This assay allows the measurement of the lipo- and hydro-philic antioxidants present in a sample. Briefly, 10 mL of cell lysate or standard (0.125–2.0 mM) was incubated in ABTS-met-Myo-PBS buffer, and the absorbance at 734 nm was monitored for 2 min. Then, the pro-oxidative insult was initiated by the addition of H_2_O_2_ (0.45 mM) and the variation in absorbance was recorded in the following 10 min of reaction. The variation in absorbance detected was compared to that obtained using the different Trolox^®^ standards (standard curve). Cell lysate TAC is expressed as micromoles Trolox equivalent/mg protein tested. 

### 2.6. Wound Closure Assay

The “wound closure assay” evaluates the ability of myoblasts to replicate and migrate in a culture plate under different experimental conditions [49]. Briefly, C2C12 myoblasts (2 × 10^3^/cm^2^) were cultured in proliferative conditions in 6-well plates until approximately 50% confluence was reached. To create a discontinuity in the cell culture, a vertical wound (1 mm) down through the cell monolayer was created by using a 1000 μL pipette tip in a sterile environment, pressing firmly against the tissue culture plate and taking care not to scratch the support. Afterward, culture media and cell debris were carefully removed, and fresh media were added under sterile conditions. Then, an initial picture was taken (named time = 0), and after a recovery period of two hours, cells were treated with MOLE (1:100), H_2_O_2_ (0.3 mM) and the combined treatments for the subsequent 24 h. At the end of the experimental time, the width of the wound was measured in triplicate and photographed. The distance between the sides of the wound were measured (in triplicate) using ImageJ software (version 1.51, Rasband, W.S., ImageJ, U.S. National Institutes of Health, Bethesda, MD, USA) to evaluate wound closure after different treatments.

### 2.7. Glutathione Homeostasis 

C2C12 glutathione homeostasis, including reduced (GSH) and oxidized (GSSG) glutathione levels and the GSH/GSSG ratio, was quantified by a DTNB–glutathione reductase recycling assay, as previously described [50]. Briefly, an adequate number of cells (>10^7^ cell collected) were suspended in (1:1) (*v*/*v*) mL 5% sulfosalicylic acid (SSA). The cells were then lysed (by freezing and thawing the samples three times) and then immediately centrifuged at 10,000× *g* for 5 min at +4 °C. The resulting deproteinized supernatant was analyzed for total glutathione content (tGSH). Oxidized glutathione (GSSG) was selectively measured in samples in which reduced glutathione (GSH) was masked by the pretreatment with 2-vinylpyridine (2%) [43]. During the assay, 10 mL of the sample was mixed with the reaction buffer, which consisted of 700 µL NADPH (0.3 mM), 100 µL DTNB (6 mM), and 190 µL H_2_O. The reaction was started by adding 2.66 U/mL glutathione reductase. The variation in absorbance recorded at 412 nm by the TNB stoichiometric formation was noted and compared to those obtained by using glutathione standards. The results were then normalized to protein content.

### 2.8. Statistical Analysis

The Kolmogorov–Smirnov test was used to test the normal distribution of the data. For all experiments, data are expressed as the means ± standard deviation (SD) of three independent experiments, each performed in triplicate. To evaluate the statistically significant differences of the data among the groups for each variable tested, one-way ANOVA for repeated measures followed by Bonferroni post hoc analyses were performed. SPSS for Windows (Version 17.0; SPSS Inc., Chicago, IL, USA) was used and statistical significance was set at *p* < 0.05. The comparisons between the untreated controls and the control vehicles showed no statistical differences for all variables tested [29,43].

## 3. Results

### 3.1. MOLE Metabolomic Fingerprint

Table 1 shows the relative percentages of metabolite groups identified in the MOLE metabolomic fingerprint. Glucosinolates (GLs) made up the largest share at 42.1%, followed by flavonoids (18.8%), as well as phenolic acids (4.5%) and other metabolites (34.6%; among them, amino acids, vitamins, and fatty acids). The table shows the three most represented bioactive molecules for each identified category.

Notably, the most intense peaks corresponded to GLs. Due to the absence of reference standards for some GLs and their associated MS/MS spectral information, an alternative identification approach was necessary. Glucosinolates are thioglucoside compounds containing a sulfated aldoxime moiety and a variable side chain derived from amino acids. This unique chemical structure produces diagnostic fragment ions in MS/MS spectra. Specifically, a sulfated glucose moiety (fragment at 259.0122 *m*/*z*) and a sulfate group (fragment at 96.9601 *m*/*z*) were readily identifiable in the spectra [33,51]. Using this method, the following glucosinolates were identified from the ion chromatogram (XIC), MS, and SWATH MS/MS spectra, with the following retention times (tR) and relative percentages: glucosoonjnain (tR: 3.8), glucomoringin (tR: 4.2), sinalbin (tR: 4.3), 4-O-acetyl-rhamnopyranosyloxybenzylGS (tR: 6.0, 6.4, 7.7), and 4-O-acetyl-glucopyranosyloxybenzylGS (tR: 5.3, 7.0).

### 3.2. Effect of Hydrogen Peroxide Treatment on C2C12 Myoblast Viability and Total Antioxidant Capacity

**Figure 1:**
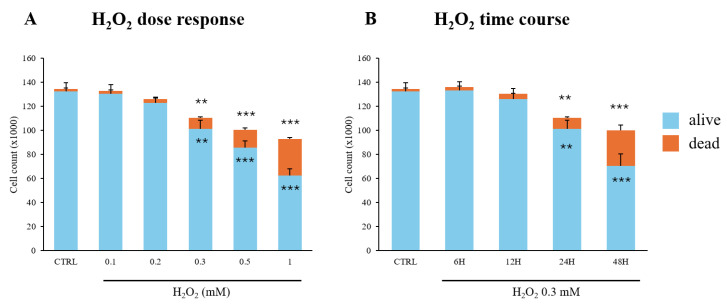
C2C12 myoblast viability under oxidative insult. (**A**) C2C12 myoblasts were treated with varying concentrations of H_2_O_2_ (0.1–1 mM) for 24 h to assess dose-dependent effects; (**B**) C2C12 myoblasts were treated with H_2_O_2_ 0.3 mM for different durations (6–48 h) to evaluate time-dependent effects. Cell viability was assessed using the Trypan blue exclusion assay. Results are expressed as the number of cells (×1000) counted. Data presented are the mean ± SD of three different experiments, each performed in triplicate. Statistical analyses were conducted using one-way ANOVA followed by Bonferroni’s multiple comparisons test. ** *p* < 0.01; *** *p* < 0.001 vs. CTRL.

C2C12 myoblast viability was assessed using the Trypan blue exclusion assay, which distinguishes dead from living cells through direct cell counting under an inverted microscope. For the dose-dependent experiments, cells were treated with a range of H_2_O_2_ concentrations (0.1–1 mM) for 24 h. We observed a dose-dependent effect of H_2_O_2_ treatment, indicated by a decrease in the number of viable cells and a concomitant rise in dead cells (Figure 1A). A concentration of 0.3 mM of H_2_O_2_ was then selected for all subsequent experiments.

In the time-course (6–48 h) experiments, a statistically significant decrease in viable cells, along with an increase in dead cells, was noted after 24 h of treatment (Figure 1B). 

**Figure 2:**
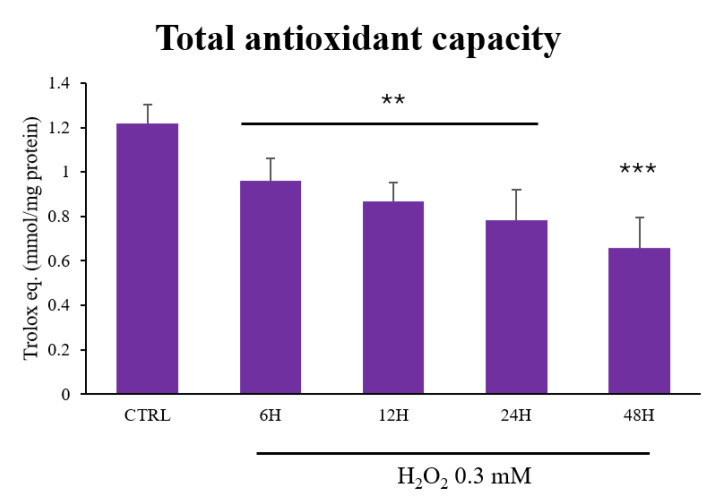
H_2_O_2_ treatment and total antioxidant capacity. C2C12 myoblasts were treated with H_2_O_2_ 0.3 mM for different durations (6–48 h). The total antioxidant capacity (TAC) of C2C12 myoblasts is expressed as micromoles of Trolox equivalent/mg protein tested. Data presented are the mean ± SD of three different experiments, each performed in triplicate. Statistical analysis was conducted using one-way ANOVA followed by Bonferroni’s multiple comparisons test. *** *p* < 0.01 and ** *p* < 0.05 compared to control (CTRL).

To evaluate whether the H_2_O_2_ treatment altered the antioxidant capacity in the myoblasts, we tested the time-dependent effects of 0.3 mM H_2_O_2_ administration on cellular total antioxidant capacity (TAC) over a period of 6 to 48 h. We observed a statistically significant decrease in TAC values over time (*p* < 0.05), with a reduction of up to 45% after 48 h of treatment (*p* < 0.01, Figure 2).

### 3.3. Effect of MOLE Supplementation on C2C12 Myoblast Viability

**Figure 3:**
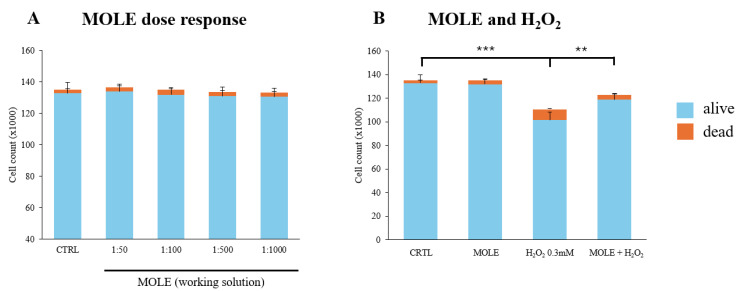
Effect of MOLE and H_2_O_2_ in single and combined treatments on C2C12 myoblast viability. (**A**) C2C12 myoblasts were treated with different MOLE dilutions (1:50–1.1000 of stock solution) for 24 h; (**B**) C2C12 myoblasts were treated single and combined treatments of H_2_O_2_ 0.3 mM and MOLE (1:100 working solution) for 24 h. Cell viability was assessed using the Trypan blue exclusion assay. Results are expressed as the number of cells (×1000) counted. Data presented are the mean ± SD of three experiments, each performed in triplicate. One-way ANOVA was performed, followed by Bonferroni’s multiple comparisons tests for panel (**A**) and (**B**), respectively. *** *p* < 0.001 vs. CTRL; ** *p* < 0.01 vs. H_2_O_2_.

To evaluate the effect of MOLE administration on C2C12 myoblasts, cells were treated with a range of working solutions (1:50–1:1000 stock solution dilutions) for 24 h. No statistically significant effect of MOLE administration was observed (Figure 3A).

For the subsequent experiments, we selected H_2_O_2_ at 0.3 mM and a MOLE dilution of 1:100. Among the different working solutions of MOLE, we chose the 1:100 dilution because it has been shown to have important biological effects in in vitro experiments on muscle cells [29,43,44,45].

We found that hydrogen peroxide exposure induced a decrease in the total number of cells (24% reduction, *p* < 0.001) and a fourfold increase in the number of dead cells (*p* < 0.001) compared to control cells (Figure 3B). Notably, MOLE administration significantly mitigated the harmful effects induced by hydrogen peroxide (*p* < 0.01, Figure 3B).

### 3.4. Effect of MOLE on C2C12 Myoblast Migrative Ability

To investigate the effects of our treatments on the migratory ability of myoblasts, C2C12 grown under proliferative conditions (<50% confluence) was subjected to a scratch wound assay, and wound closure was measured after 24 h of recovery [49]. Briefly, in the wound closure assay, a wound of 1 mm was created in the culture plate before each treatment, and after 24 h, the width of the wound was measured in the control and treated cell samples. 

**Figure 4:**
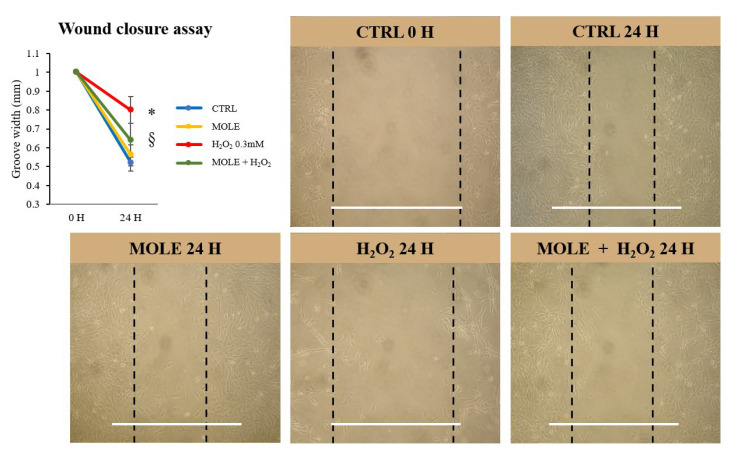
Wound closure assay. A 1 mm sulcus was created in the cell culture under sterile conditions before the experimental protocols were carried out. Cells were treated with MOLE (1:100 working solution), H_2_O_2_ 0.3 mM, or combined treatments, as well as a vehicle control, for 24 h. The sulcus width was then measured in both the control and treated cell samples. On the graph, the effects of MOLE, H_2_O_2_, and combined treatment on sulcus width are shown. The images shown are representative of the experiments and all treatments performed. The white lines indicate the 1 mm measurement of the groove created before the treatments. Data and representative images presented are the mean ± SD of three experiments, each performed in triplicate. One-way ANOVA was performed followed by Bonferroni’s multiple comparisons tests. * *p* < 0.05 vs. CTRL; § *p* < 0.05 vs. H_2_O_2_.

After 24 h, reductions in wound width of 48% and 44% were observed for the CTRL and MOLE samples, respectively (Figure 4). After a single 24 h H_2_O_2_ treatment, a minor reduction was observed compared to the control (20% reduction, *p* < 0.05). 

The combined MOLE + H_2_O_2_ treatment showed that the presence of MOLE enhanced the cells’ ability to close the wound, resulting in a 36% reduction in wound width compared to H_2_O_2_ alone (*p* < 0.05, Figure 4). 

### 3.5. Effect of MOLE on C2C12 Myoblast Redox Status and Total Antioxidant Capacity

The hydrogen peroxide-induced perturbation of the redox state and the effect of MOLE were evaluated by analyzing glutathione homeostasis. In particular, the GSH/GSSG ratio is a well-known marker of redox status. We found statistically significant differences in the GSH/GSSG ratio between the different treatments of the C2C12 myoblasts. The H_2_O_2_ treatment induced a decrease in total glutathione levels (tGSH, 13% *p* < 0.01), an increase in GSSG levels (152% *p* < 0.05), and a decrease in the GSH/GSSG ratio (51% *p* < 0.05) compared to the control (Table 2). No statistically significant differences in glutathione homeostasis were found after the MOLE treatments. Interestingly, the combination of MOLE with H_2_O_2_ significantly prevented the H_2_O_2_-induced increase in oxidized glutathione (*p* < 0.01, Table 2) and the decrease in the GSH/GSSG ratio (*p* < 0.01, Table 2).

**Figure 5:**
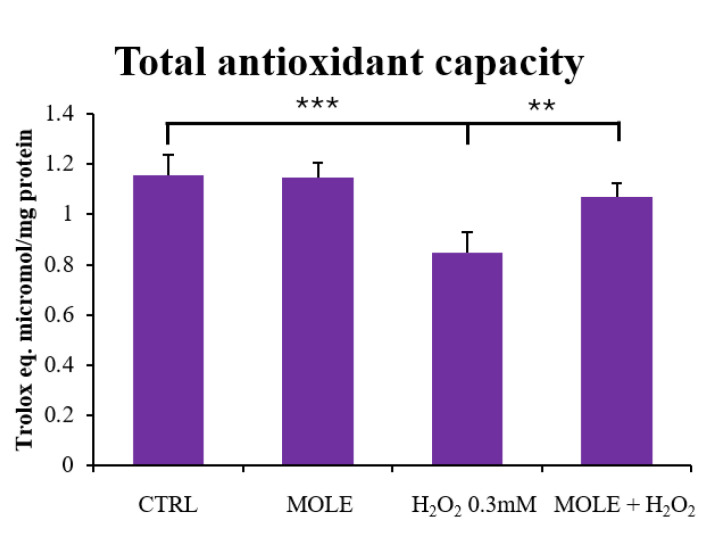
Effect of MOLE and H_2_O_2_ on total antioxidant capacity in C2C12 myoblasts. C2C12 myoblasts were treated with H_2_O_2_ 0.3 mM, MOLE (1:100 working solution), and combined treatments for 24 h. The total antioxidant capacity (TAC) of cell lysates was measured and is expressed as micromoles of Trolox equivalent/mg protein. Data are presented as the mean ± SD of three independent experiments, each performed in triplicate. Statistical analysis was conducted using one-way ANOVA followed by Bonferroni’s multiple comparisons test. *** *p* < 0.001 vs. CTRL; ** *p* < 0.01 vs. H_2_O_2_.

The analysis of total antioxidant capacity revealed a statistically significant reduction following 24 h of H_2_O_2_ treatment (*p* < 0.001, Figure 5). The combined treatment with MOLE significantly reversed this decrease (*p* < 0.01, Figure 5).

## 4. Discussion

Oxidative stress is a condition that can seriously compromise cellular functionality, primarily through the disruption of cellular antioxidant homeostasis. One potential strategy to counteract oxidative stress is the use of bioactive molecules with antioxidant properties. This approach is particularly important in areas that focus on muscle metabolism, muscle function, and sports performance.

In this context, to meet the increasing demand for nutritional interventions targeting oxidative stress, many studies have focused on strategies aimed at enhancing physical performance, and especially reducing fatigue and increasing exercise endurance. These conditions are closely linked to elevated oxidative stress levels [24,52,53,54].

Skeletal muscle tissue is particularly vulnerable to the harmful effects of oxidative stress due to its high oxygen consumption. During physical exercise, especially in intense or unaccustomed activities, muscle contractions are often accompanied by high ROS production due to increased metabolic activity. This activity can lead to oxidative stress and potential myofibrillar damage, which is evidenced by elevated biomarkers of oxidation in both skeletal muscle and blood [55,56]. 

To preserve muscle function and protect cells from excessive ROS exposure, the use of antioxidants has become a common strategy. Along these lines, appropriate antioxidant use has been shown to effectively balance the oxidant–antioxidant ratio in most physiopathological conditions [52,57,58].

Among the natural extracts gaining recent popularity, *Moringa oleifera* extract has shown promising results in muscle cell models as a supplement capable of counteracting oxidative stress. We previously demonstrated that *Moringa oleifera* leaf extract (MOLE) shows dose-dependent total antioxidant capacity in cell-free systems, and this indicates that the efficacy of the leaf extract depends on the concentration of all the antioxidant molecules present [43,44]. Moreover, MOLE has been shown to activate the antioxidative metabolism through the SIRT1-PPAR and Nrf2 pathways (including its target gene, HO-1), both of which are regulators of cellular resistance to oxidants in C2C12 myotubes. 

MOLE also enhanced cellular antioxidant capacity by improving glutathione redox homeostasis and increasing antioxidant enzyme activities in C2C12 myotubes [29,43]. These effects were significant in protecting myotubes under oxidative insult conditions induced by H_2_O_2_ treatment [44]. 

Muscle microtrauma commonly occurs after exercise and is normally associated with elevated inflammatory processes and reactive oxygen species (ROS) production, which may lead to the secondary damage of myofibers [59]. In response to such injury, satellite cells are activated, exiting their quiescent state, entering the cell cycle, and proliferating. These cells give rise to new cells that either return to quiescence (self-renewal) or proceed to terminal differentiation. During this phase, the cells become myoblasts, which fuse with damaged myofibers to form new muscle fibers or, alternatively, assist in growing old uninjured fibers [2,60,61]. 

In this study, we focused on myoblasts under oxidative insult and supplementation with a mix of molecules with antioxidant action (for example, polyphenols, phenolic acids, etc.) because the efficiency of myoblasts is a key factor for optimal muscle regeneration.

Recently, studies utilizing antioxidant compounds to mitigate excess ROS production in skeletal muscle during the repair process following myotrauma have provided evidence of enhanced muscle recovery. This recovery is characterized by the upregulation of the myogenic potential, viability, maintenance, and activity of satellite cells in both in vitro and in vivo experimental models [62,63,64].

In this study, we employed C2C12 myoblasts, which are derived from satellite cells and serve as a robust model for investigating muscle proliferation and differentiation in vitro [65,66,67]. C2C12 cells are widely used to study muscle regeneration due to their capacity, under appropriate stimuli, to transition from a proliferative phase into differentiated myofibers, resembling the behavior of satellite cells.

The present study demonstrated that mild, non-cytotoxic H_2_O_2_ treatment caused growth inhibition, perturbation in redox status, and a decrease in total antioxidant capacity in C2C12 myoblasts. These negative effects induced by oxidative insults can seriously compromise the metabolism of satellite cells and, consequently, the regenerative capacity of muscle fibers. Given that antioxidant supplementation is one strategy to counteract these effects, in this work we also evaluated the effect of *Moringa oleifera* leaf extract (MOLE) on the vitality and proliferation, as well as the redox state, of myoblasts subjected to oxidative insult. 

Interestingly, we found that MOLE supplementation had a significant effect in counteracting the harmful effects of hydrogen peroxide by restoring redox status and improving the vitality and proliferation of C2C12 myoblasts.

*Moringa oleifera* Lam. is commonly referred to as the “miracle tree” due to its numerous beneficial properties, which have significant implications for both therapeutic and nutritional applications. In fact, different parts of the plant, such as the leaves, seeds, and roots, are used by different populations in the world to treat various pathological conditions. For example, *Moringa oleifera* preparations are used to support cardiovascular health and regulate blood glucose levels, exhibiting antioxidant, anti-inflammatory, and potential anti-cancer properties [68,69,70,71,72,73,74,75,76]. 

In particular, the leaves of *Moringa oleifera* are highly nutritious, making it easy to incorporate them into one’s daily diet to benefit from their therapeutic and nutritional properties. Leaves can be eaten fresh, such as in salads, or dried to produce moringa powder and added to dishes. However, the powder should not be heated above about 40 °C so as not to denature the myrosinase. Many valuable bioactive molecules are present in fresh leaves and many of these molecules are retained in significant quantities even after preparation for food consumption [35,77]. 

First, we conducted a metabolomics analysis of the ethanolic extract of the leaves. The highly sensitive high-resolution LC-MS/MS technique [78], specifically using a ZenoTOF 7600 mass spectrometer, significantly enhanced the detection and confident identification of a higher number of bioactive molecules in the MOLE extracts by combining the Zeno-DDA and Zeno-SWATH approaches [45]. This analysis identified the presence of many bioactive molecules, including glucosinolates, flavonoids, phenolic acids, and other metabolites. Among them were amino acids, vitamins, and fatty acids with important nutritional value.

Most of these molecules show antioxidant properties, which is further supported by their effectiveness in mitigating the oxidative damage induced by H_2_O_2_ in the cell culture model. The antioxidant capacity of the leaf extract is likely attributable to the collective properties of the various bioactive molecules present in Brassicalean extracts, particularly in Moringa oleifera, as previously documented [79,80,81,82,83,84,85]. Isothiocyanates, polyphenols, flavonoids, and phenolic acids are known to exhibit antioxidant abilities, either preventing hyperperoxide decomposition or inactivating lipid free radicals [86,87,88]. Glucosinolates (GSLs) are typical of the order Brassicales (e.g., Brassicaceae, Capparaceae, Caricaceae, etc.), and are known for their health-promoting and antioxidative properties, which are mediated by the bioactive molecules present [89,90,91,92,93]. Glucosinolates are hydrolyzed by myrosinase activity into isothiocyanates, such as benzyl isothiocyanate, phenyl isothiocyanate, and sulforaphane [94]. These compounds are potent activators of the enzymatic antioxidant system, primarily by upregulating Nrf2-mediated gene induction, which includes increases in the mRNA levels of GCLC, NQO1, and HO-1, as well as elevated HO-1 protein levels. Additionally, the p38 MAPK signaling pathway [95], known for its role in regulating Nrf2 phosphorylation and nuclear translocation, is also activated by isothiocyanates [96,97]. 

Isothiocyanates support mitochondrial function and help maintain protein integrity under oxidative stress. It has been demonstrated that sulforaphane, one of the most studied isothiocyanates, is able to reduce muscle damage and inflammation in individuals subjected to oxidative stress induced by physical exercise [98]. Furthermore, it alleviates muscle soreness by upregulating Nrf2-target NQO1 expression [99]. 

Isothiocyanates also exhibit Nrf2-independent effects, including the inhibition of mitochondrial fission and the modulation of the mTOR pathway [100]. 

Moreover, glucosinolates, in addition to regulating oxidative stress levels, influence inflammation, cell proliferation, cell-cycle arrest, apoptosis, angiogenesis, and invasion, as well as modulating the activity of cancer cells [101,102,103]. 

It should be noted that the metabolomic analysis detected only very small amounts of isothiocyanates compared to glucosinolates in the MOLE sample, including 4-[(α-Lrhamnosyloxy) benzyl]isothiocyanate, 4-[(2′-O-acetyl-alpha-L-rhamnosyloxy)-benzyl]-isothiocyanate, 4-[(3′-O-acetyl-alpha-L-rhamnosyloxy)-benzyl]-isothiocyanate, and 4-[(4′-O-acetyl-alpha-L-rhamnosyloxy)-benzyl]-isothiocyanate (derived from glucomoringin, 4-O-acetylrhamnopyranosyloxybenzyl-GS 1, 4-O-acetylrhamnopyranosyloxybenzyl-GS 2, and 4-O-acetylrhamnopyranosyloxybenzyl-GS 3, respectively). However, as a limitation, we cannot exclude their production from glucosinolate metabolism in myoblasts.

Polyphenols, including subclasses such as flavonoids, flavanols, and phenolic acids [104,105,106], are well known for their antioxidant properties, which involve scavenging and neutralizing free radicals [107]. They mitigate oxidative stress and cellular damage induced by reactive oxygen species (ROS) [108], modulate the activity of endogenous antioxidant enzymes such as SOD, CAT, and GPx [107,109], and regenerate other antioxidants, such as vitamins C and E [110]. Moreover, these molecules are able to activate specific cellular defense mechanisms and to promote the repair of damaged molecules. This has a significant consequence of enhancing cellular resilience against oxidative damage [111]. 

Flavonols, such as kaempferol and quercetin, are well-known potent antioxidants capable of inhibiting lipid peroxidation and enhancing endogenous antioxidant defenses through the activation of enzymes like superoxide dismutase (SOD) and catalase (CAT) [112]. Notably, quercetin protects myotubes against tumor necrosis factor (TNF)-induced muscle atrophy under obese conditions by inducing Nrf2-mediated heme oxygenase-1 (HO-1) expression while inhibiting NF-kB activation [113]. Furthermore, it has been demonstrated that quercetin is able to promote mitochondrial biogenesis in skeletal muscles. This has an important impact on improving cellular mitochondrial function, protein content, enzyme activity, and respiratory function [23,114,115,116,117].

Additionally, catechin flavonoids present in *Moringa oleifera*, such as epicatechin and epigallocatechin, are recognized for their antioxidant properties [118,119,120,121,122]. Polyphenols also play a crucial role in maintaining cellular proliferative capacity under oxidative stress [123,124,125]. Among these, phenolic acids, including hydroxybenzoic acids (e.g., gallic acid) and hydroxycinnamic acids (e.g., caffeic acid), exhibit antioxidative properties that contribute to cellular defense against oxidative stress. Specifically, gallic acid and caffeic acid scavenge free radicals, inhibit lipid peroxidation, and preserve cellular health by modulating antioxidant enzyme activity [126,127,128]. 

While *Moringa oleifera* leaves (MOLE) contain various bioactive molecules with antioxidant action, it is important to recognize that the biological effects of the extracts likely stem from the synergistic effects of the entire mixture of bioactive compounds rather than a single component [29,43,45]. Key molecules in MOLE include glucosinolates (glucomoringin and 4-O-acetylrhamnopyranosyloxybenzyl-GS) and other important molecules, such as flavonoids (e.g., isoquercitrin, astragalin, and rutin), phenolic acids (e.g., chlorogenic acid), and lipids (e.g., omega-3 alpha-linolenic acid). These compounds contribute to the antioxidant protective action of MOLE either independently [129,130,131] or through synergistic effects [29,43,45].

Interestingly, treatment with MOLE increased proliferation in H_2_O_2_-treated cells, as evidenced by accelerated recovery in the wound scratch assays compared to the control cultures. This assay simulates a damaging situation, and myoblasts at the injury site are evaluated for their migration and proliferation capacity [49]. It mimics the degeneration/inflammation phase preceding muscle regeneration, during which satellite cells/myoblasts migrate to the injury site, proliferate, and begin the differentiation process.

As discussed previously, the production of reactive oxygen or nitrogen species following increased metabolic rate/muscle activity and the increase in inflammatory events following mechanical stress/fiber rupture and inflammatory reaction create oxidative damage, as widely evidenced in the literature with the increase in markers such as malondialdehyde (MDA), thiobarbituric acid reactive substances, and protein carbonyls, which are detected in the blood or muscle cells [132,133].

At the same time, no meaningful differences in antioxidant enzyme activity are reported in the blood [132]. However, in whole muscle lysates, the GSH/GSSG ratio is frequently decreased after the oxidative insult, whereas the levels of antioxidant enzyme activity only slightly increase, most notably when compared with markers of oxidative stress/damage [132,133].

The presence of MOLE in combination with H_2_O_2_ facilitated the restoration of wound closure capability. This was attributed to the improvement of the redox state of myoblasts, as demonstrated by the evaluation of the GSH/GSSG ratio and the analysis of total antioxidant capacity, thereby partially restoring the resting condition negatively affected by the oxidative insult from H_2_O_2_. Under our experimental conditions, the GSSG content was higher than that found in vivo [134]. However, these are values that are always found using the experimental model of C2C12 muscle cells.

The evaluation of the intracellular TAC showed that the treatment with MOLE, due to the presence of bioactive molecules with antioxidant action, counteracted the decrease in antioxidant capacity observed after the treatment with hydrogen peroxide.

However, in the analysis of the data, the experimental limitations that this method entails must be taken into consideration [135]. Certainly, more in-depth studies on the modulation of the level of molecules with intracellular antioxidant potential are warranted in the future.

Overall, our results provide important insights into the regeneration process of skeletal muscle, which is a complex event mediated by satellite cells. Maintaining the correct redox balance is crucial for the proliferative and regenerative capacity of myoblasts. A detailed understanding of these mechanisms is essential for comprehending pathological conditions that lead to skeletal muscle degeneration and for identifying targeted therapeutic strategies. In this context, *Moringa oleifera* leaf extract emerges as a protective supplement against oxidative stress and could help muscle regeneration processes.

## 5. Conclusions

This study demonstrated that oxidative stress induced by H_2_O_2_ exposure disrupted the redox balance and reduced cell proliferation in C2C12 myoblasts. The combined treatment with MOLE exhibited antioxidant effects by significantly restoring total cellular antioxidant capacity, improving the GSH/GSSG ratio, and enhancing cell viability, leading to the restoration of the proliferative capacity of the cells. Thus, we conclude that MOLE serves as a protective supplement against oxidative stress and can support muscle regeneration processes, making it a significant aid in conditions related to reduced muscle regenerative capacity. Further studies on the molecular mechanisms underlying the effects of MOLE, particularly in degenerative conditions, are warranted.

## Figures and Tables

**Table 1:** Relative amounts of constituents of MOLE ^1^.

**Glucosinolates (relative %)**	**42.1 ± 0.3**
4-O-acetyl-rhamnopyranosyloxybenzyl-GS	31.6 ± 0.4
Glucomoringin	10.3 ± 0.2
4-O-acetyl-glucopyranosyloxybenzyl-GS	0.2 ± 0.1
**Flavonoids (relative %)**	**18.8 ± 0.2**
Isoquercitrin	6.3 ± 0.3
Astragalin	4.5 ± 0.2
Rutin	2.4 ± 0.2
**Phenolic acids (relative %)**	**4.5 ± 0.1**
Cryptochlorogenic acid	2.2 ± 0.1
Chlorogenic acid	1.8 ± 0.2
Quinic acid	0.3 ± 0.1
**Others (relative %)**	**34.6 ± 0.4**
Alpha-linolenic acid	19.2 ± 0.4
Linoleic acid	9.2 ± 0.3
Pantothenic acid	1.6 ± 0.2

^1^ Metabolomic analysis was conducted on the MOLE working solution. The relative percentages of the most abundant biomolecules in MOLE are categorized into different groups: glucosinolates, flavonoids, phenolic acids, and others, with their respective percentages for each category. The table shows the three most represented bioactive molecules for each identified category. The data presented are the mean ± SD of three different extractions, each tested in triplicate.

**Table 2:** Glutathione homeostasis evaluation ^1^.

**Total glutathione (tGSH)**	
CTRL	206.5 ± 7.5
MOLE	222.9 ± 7.8
H_2_O_2_ 0.3 mM	181.6 ± 3.3 **
MOLE + H_2_O_2_	199.4 ± 13.5
**Oxidized glutathione (GSSG)**	
CTRL	20.5 ± 4.3
MOLE	25.4 ± 0.8
H_2_O_2_ 0.3 mM	31.2 ± 1.5 **
MOLE + H_2_O_2_	26.6 ± 0.4 §
**Reduced to oxidized glutathione ratio (GSH/GSSG)**	
CTRL	9.5 ± 1.9
MOLE	7.8 ± 0.1
H_2_O_2_ 0.3 mM	4.8 ± 0.4 *
MOLE + H_2_O_2_	6.5 ± 0.4 §

^1^ Total glutathione (tGSH) nanomol/mg protein, oxidized glutathione (GSSG, nanomol/mg protein), and the reduced-to-oxidized glutathione ratio (GSH/GSSG) were evaluated in C2C12 myoblasts after 24 h in the presence of MOLE (1:100 working solution), hydrogen peroxide (H2O2, 0.3 mM), and combined treatments. Data are presented as the mean ± SD of three independent experiments, each performed in triplicate. Statistical analysis was conducted using a two-way ANOVA followed by Bonferroni’s multiple comparisons test. * *p* < 0.05, ** *p* < 0.01 vs. CTRL; § *p* < 0.01 vs. H2O2.

## Data Availability

Data are contained within the article.

## Abbreviations

Moringa oleifera leaf extract (MOLE), reactive oxygen species (ROS), glucosinolates (GLs), reduced (GSH) and oxidized (GSSG) glutathione, reduced-to-oxidized glutathione ratio (GSH/GSSG), total antioxidant capacity (TAC).
